# Medicinal Plants Facilitate the Recovery of Diabetic Foot Ulcer by Regulating Macrophages: Latest Evidence and Insights

**DOI:** 10.2174/0115733998332536241205164427

**Published:** 2024-12-23

**Authors:** Hongping Mo, Zhen Ye, Xudong Wen, Tao Pan, Zejun Chen, Qiaobo Ye

**Affiliations:** 1 Internal Medicine Department, Chengdu University of Traditional Chinese Medicine Affiliated Hospital of Integrated Traditional Chinese and Western Medicine, Chengdu, 610059, China;; 2 School of Basic Medical Sciences, Chengdu University of Traditional Chinese Medicine, Chengdu, 611137, China

**Keywords:** Medicinal plants, diabetic foot ulcer, macrophages, wound healing, diabetic wounds, lower-limb amputations

## Abstract

Diabetic Foot Ulcer (DFU) is a major complication of diabetes that mostly affects the lower extremities, with a high incidence and recurrence rate in approximately 15% of patients with diabetes. The complexity of diabetic wounds poses a substantial challenge for clinical recovery, underscoring the need to investigate novel therapeutic approaches. Medicinal plants have been used to treat ulcers for centuries. Recently, there has been a growing focus on the development of topical preparations derived from medicinal plants that target macrophages as an adjuvant therapy for DFU. Macrophages have been identified as crucial factors in the DFU healing process. This review aims to introduce the latest evidence and insights into the role of medicinal plants in promoting DFU recovery by targeting macrophages. The molecular mechanisms underlying the preventive effects of medicinal plants on DFU primarily involve promoting M2 polarization of macrophages, inhibition of M1 polarization, and regulation of macrophage function. This review highlights the substantial potential of advancing the field of DFU management by medicinal plants and lays the groundwork for novel therapeutic interventions.

## 1 INTRODUCTION

Recently, over 463 million individuals have been diagnosed with diabetes, which is expected to reach 700 million by 2045 [1]. Diabetic Foot Ulcers (DFU), one of the most debilitating complications of diabetes, affect approximately 15% of patients with diabetes and present a significant global public health concern [2]. Furthermore, more than 60% of non-traumatic lower-limb amputations occur within the diabetic population [3]. The poor prognosis for survival among patients with DFU, with a survival rate of approximately 50% within five years even after amputation, underscores the challenges associated with DFU, which extend beyond mere health implications [4]. The high incidence and intractability of DFU contribute to escalating healthcare expenses and diminished productivity. According to a literature survey conducted by the Center for Disease Control and Prevention in the USA, the economic burden of diabetes care is increasing. A survey revealed that the direct costs associated with diabetes care amount to approximately $273 billion annually, accompanied by an additional $90 billion in indirect costs (https://www.cdc.gov/chronicdisease).

The current standard clinical care for DFU includes debridement, offloading, wound dressings, and antibiotics [5]. However, the effectiveness of these treatments is limited, resulting in a significant number of patients requiring amputation [6]. To enhance DFU healing, researchers have diligently investigated multiple alternative therapies. Stem cell-based therapy is potentially proven effective for DFU treatment, but it can not fully restore the normal anatomy and lacks long-term observation [7-10]. Hyperbaric oxygen therapy is reported to speed up DFU recovery, but complications are inevitable [11-13]. Skin substitutes have been increasingly developed, such as matrices with or without allogeneic cells and autologous cells, but a series of preclinical assessments might be required [14, 15]. In addition, a limited number of studies suggest the effectiveness of growth factors [14, 16], 3D bioprinting technologies [17], and laser therapy [18] in DFU recovery, but high-quality clinical evidence is needed.

To explore effective therapeutic strategies for DFU, a deep understanding of the underlying pathophysiological processes of the disease is warranted [19, 20]. Wound healing is a complex process that encompasses distinct yet interconnected stages, including hemostasis, inflammation, proliferation, and remodeling [21]. It relies on the coordinated efforts of various cells, including platelets, neutrophils, monocytes, macrophages, endothelial cells, keratinocytes, fibroblasts, and myofibroblasts [22]. Macrophages play critical regulatory roles as immune cells, particularly during the inflammatory phase of DFU healing [23]. In recent years, medicinal plants have been reported to promote DFU healing by regulating non-parenchymal cells, providing new clinical evidence for their use in wound healing therapy. Indeed, medicinal plants have been used for centuries owing to their therapeutic properties, which generally have fewer side effects than synthetic drugs [24]. Numerous studies have documented the wound-healing properties of medicinal plants, which could be attributed to the presence of bioactive compounds, such as alkaloids, flavonoids, terpenoids, and phenolic compounds [25]. These bioactive compounds exhibit a range of beneficial properties, including anti-inflammatory, antimicrobial, and antioxidant properties [26]. Consequently, they play crucial roles in promoting wound healing by reducing inflammation, preventing infection, and enhancing tissue regeneration. Notably, studies have reported the ability of medicinal plants to modulate macrophage activity, leading to improved wound healing outcomes [27]. For instance, Aloe vera increased the recruitment of macrophages to wound sites and enhanced their phagocytic activity [28]. Curcumin cleared debris, secreted growth factors, and recruited immune cells to the wound sites, thereby facilitating tissue repair and regeneration [29]. Similarly, *Curcuma longa* (turmeric) has been found to enhance macrophage-mediated clearance of bacteria and promote tissue regeneration [30].

The development of new medicinal plant-based products that target macrophages has emerged as a prominent area of research in DFU treatment. Aligning with this objective, this narrative review aims to provide a comprehensive summary of medicinal plants that exert therapeutic effects on DFU by modulating macrophage activity. A flowchart illustrating the literature screening process is shown in Fig. (**1**). By elucidating the potential of medicinal plants for DFU treatment, this review seeks to establish a solid foundation for their clinical application and facilitate the development of novel drugs for the prevention and treatment of DFU. This study holds significant promise for advancing the field of DFU management and opens new avenues for therapeutic interventions.

## 2 POLARIZATION OF MACROPHAGES IN DFU

### 2.1 Classification of Macrophages

Macrophages are important constituents of the mononuclear phagocytic system and are widely distributed in diverse tissues and organs. Macrophages could dynamically adapt to varying microenvironments by changing their phenotype, thus enabling them to perform distinct functions [31]. Upon stimulation by different pathogen- and damage-associated molecular patterns, macrophages undergo polarization, resulting in the emergence of two distinct types: M1 macrophages (known as classical activation pathway macrophages) and M2 macrophages (referred to as alternative activation pathway macrophages) [32].

During the inflammatory phase, recruited monocytes differentiate into M1 macrophages in response to lipopolysaccharide (LPS) and other bacterial products, interferon-gamma, tumor necrosis factor-alpha (TNF-α), and granulocyte-macrophage colony-stimulating factor [33]. These M1 macrophages release pro-inflammatory cytokines, such as interleukin (IL)-6, TNF-α, IL-1β, and inducible nitric oxide synthase (iNOS), to fight infection and promote the killing of microbial pathogens [34]. M1 macrophages regulate wound cellularity by inducing apoptosis and phagocytosis in various types of wound cells, including neutrophils, fibroblasts, and endothelial cells [27]. M2 macrophages could be classified into four subtypes: M2a, M2b, M2c, and M2d, each with distinct functions [35]. The M2a subtype, which is induced by IL-4 and IL-13, exhibits high expression of mannose receptors and enhances endocytic activity. The M2a macrophages promote cell growth, tissue repair, and Th2 immunity. M2b subtype induced by immune complexes, lipopolysaccharides or IL-1, is characterized by high secretion of inflammatory cytokines, such as TNF-α, IL-6, and IL-1, as well as anti-inflammatory cytokines like IL-10, and low secretion of IL-12. This subtype suppresses the acute inflammatory responses and supports Th2 and humoral immunity. M2c subtype, induced by glucocorticoid, IL-10, and transforming growth factor-beta (TGF-β), secretes IL-10 and proteinases while expressing low levels of major histocompatibility complex class II. This subtype inhibits the inflammatory response and promotes tissue remodeling. M2d macrophages are tumor-associated macrophages induced by a combination of toll-like receptor agonist ligands with A2 adenosine receptor agonists or IL-6. Consequently, M1 macrophages appear predominantly during the early stages of wound healing, contributing to the promotion of the wound inflammatory response, clearance of necrotic tissue and cells, and prevention of bacterial invasion by releasing inflammatory factors and chemokines [36, 37]. As the wound transitions into the post-inflammatory stage, the number of macrophages in the local wound area gradually decreases, and the remaining macrophages transform into M2 macrophages [38]. Consequently, the expression levels of inflammatory factors secreted by M1 macrophages decrease, whereas the secretion of anti-inflammatory factors increases. This reduction in the inflammatory response promotes cell proliferation and vascularization in the local wound, facilitating further wound healing (Fig. **2** and Table **1**).

### 2.2 Diabetic Microenvironment and Macrophages

Elevated blood glucose levels have a significant impact on various immune cells, particularly macrophages, which play a crucial role in repair and defense against infection [39, 40]. Disturbances in their number and phenotypes could ultimately lead to delayed wound healing. Studies have indicated that the diabetic microenvironment triggered an increased production of hematopoietic stem cells in the bone marrow, leading to higher levels of circulating blood monocytes and an increased number of macrophages that exhibited M1-like characteristics. In chronic wounds, approximately 80% of the cells at the wound edge consist of pro-inflammatory M1 macrophages [41]. These macrophages secrete numerous proteases, including matrix metalloproteinase, which selectively degrade extracellular matrix (ECM) proteins and remove the newly formed ECM. Consequently, bioactive peptides that act as chemical attractants or modulate immune cell activity are generated, all of which negatively affect wound healing. Furthermore, hyperglycemia impairs the phagocytic function of macrophages, reducing their ability to clear infections and limiting the accumulation of neutrophils within wounds. Neutrophils are crucial for proper healing, and their reduced presence exacerbates impaired healing observed in diabetes. Additionally, the transformation of macrophages from a pro-inflammatory to an anti-inflammatory phenotype, which is a necessary step for wound repair, is delayed in DFU. This delay is likely due to the high levels of advanced glycation end-products present in the wound and the reduced phagocytic capacity of macrophages. The imbalance in macrophage phenotypes and the compromised repair state caused by the diabetic microenvironment result in a persistent state of inflammation in diabetic wounds. This chronic inflammation hamper the pro-healing activities of keratinocytes, fibroblasts, and endothelial cells, which are essential for proper wound repair. Ultimately, these factors collectively contribute to delayed wound healing observed in individuals with diabetes.

### 2.3 Molecular Mechanisms of Macrophage Polarization

M1 macrophages are the predominant cells during the initial phases of wound healing, releasing pro-inflammatory cytokines and exhibiting strong cytotoxic and anti-proliferative effector functions [42]. Therefore, the M1 macrophages induce a persistent inflammatory response that might lead to tissue damage [43]. In M1 polarization, nuclear factor-kappa B (NF-κB) is activated by LPS and controls the expression of inflammatory cytokines [44]. The p65 and p60 heterodimers within the NF-κB complex are generally accepted as pro-inflammatory constituents [45]. In addition, by encouraging the autocrine cytokines TNF-α and IL-1β to maintain NF-κB activity, LPS supports the feed-forward signaling of NF-κB [46]. An essential transcription factor for M1 polarization is STAT (signal transducer and activator of transcription) 1 and its STAT family members, such as STAT2, STAT4, and STAT5. During the wound healing process, M1 macrophages transition to an M2 phenotype, which helps to mitigate tissue damage caused by inflammation. As the healing progresses, M2 macrophages become predominant, playing a crucial role in resolution and tissue repair [47]. M2 macrophages further activate fibroblasts for collagen deposition, angiogenesis, and wound contraction by releasing growth factors and anti-inflammatory cytokines [48]. In contrast to the above STAT family members, STAT3 and STAT6 coordinate M2 polarization [49]. Peroxisome proliferator-activated receptor gamma, activated by IL-4 and IL-13, also contribute to M2 polarization [50].

Given the current challenges associated with poor healing of DFU and the significant role that macrophage polarization plays in the healing process, it is especially important to elucidate the mechanisms of polarization and to regulate macrophage polarization rationally [51-53].

## 3 ROLE OF MEDICINAL PLANTS IN PROMOTING THE PREHEATING MACROPHAGE PHENOTYPE-MEDIATED HEALING OF DFU

The current understanding of dysregulation of macrophage polarization in the context of DFU revolves around the concept that high blood glucose levels lead to increased expression of inflammatory cytokines, which, in turn, promote the M1 macrophage phenotype. Thus, it appears logical to assume that addressing the primary issue of hyperglycemia would effectively mitigate the initial problem within this challenging cascade. However, despite advancements in new drugs and therapies aimed at improving blood glucose control in patients with diabetes, only 60% of individuals achieved the recommended A1c level of less than 7.0% [54]. Therefore, it is crucial to explore alternative therapies that target the sustained inflammatory phenotype to facilitate DFU healing, in addition to addressing the diabetic condition itself. A detailed graphical representation of multiple therapeutic targets involving medicinal plants or plant secondary metabolites in DFU management is presented in Fig. (**3**). These potential therapeutic targets offer promising avenues for intervention and improvement of DFU healing outcomes (Tables **2** and **3**).

### 3.1 Natural Products that Inhibit the Polarization of M1 Macrophages

Macrophage M1 polarization plays a pivotal role in the pathogenesis of inflammatory diseases, including DFU. Recent studies have revealed the potential of certain botanical agents in treating DFU by effectively inhibiting M1 macrophage polarization. ON101, the first approved macrophage regulator for the topical treatment of DFU in Taiwan [55], was formulated using specific ratios of identified and defined fractions from *Plectranthus amboinicus* and *Centella asiatica*. An international multicenter Phase III randomized clinical trial demonstrated that ON101 exhibited superior efficacy compared to absorbent dressing alone in the treatment of DFU, showing consistent efficacy across all participants enrolled in the study [56]. Lin *et al*. used two diabetic mouse models (high-fat diet-induced diabetes and db/db mice) to confirm the accelerated diabetic wound-healing properties of ON101. ON101 effectively attenuated the polarization of M1 macrophages and promoted the enrichment of M2 macrophages [57]. Notably, when ON101 was administrated during the polarization progression of M1- macrophages in tumor-promoting-monocytes isolated from peripheral blood, it directly inhibited M1 polarization by suppressing the expression of cluster of differentiation CD 80 and cluster of CD 86. Additionally, quantitative real-time PCR verification showed that ON101 downregulated M1-related chemokines (chemokine (C-X-C motif) ligand 9, CXCL10, and chemokine (C-C motif) ligand 12) and cytokines (IL-6, IL-1β, and TNF-α) while upregulating chemokines, including CCL2 and CCL3, which possess M2 macrophages-recruiting abilities. These findings suggest that ON101 treatment alleviates M1-related inflammation and creates a favorable microenvironment for monocytes and macrophages.

The medicinal herb *Angelica dahurica* is known for its anti-inflammatory, proangiogenic, and cell-stimulating properties, making it a widely used treatment for diabetes and its complications, such as DFU [58]. It has been demonstrated that *Angelica dahurica* accelerated wound healing in db/db mice *via* the HIF-1α/PDGF-β signaling pathway [59]. Hu *et al.* conducted *in vivo* experiments and a network pharmacology study, confirming the effect of *Angelica dahurica* on diabetic wounds by regulating the polarization of M1 and M2 macrophage subtypes [60]. Additionally, Chao *et al.* demonstrated that *Angelica dahurica* and *Rheum officinale* significantly decreased the expression of nuclear factor kappa- light-chain-enhancer of activated B cells in the wound skin tissue of diabetes mellitus rats [61]. Moreover, a recent study reported that phellopterin, a monomeric compound from *Angelica dahurica*, exerted an anti-inflammatory effect that facilitated diabetes-associated cutaneous wound healing *via* silent information regulator 2 homolog 1 [62].

Resveratrol, a compound derived from plants, such as grapes, has demonstrated its ability to promote angiogenesis and suppress inflammation in DFU [63]. However, topical formulation of resveratrol poses challenges owing to its physicochemical properties and UV-dependent isomerization [64]. Thus, recent research has focused on utilizing polymeric delivery systems, advanced biomaterials, and nanotechnology to optimize the therapeutic outcomes of resveratrol treatment for DFU [65]. In line with this, Zhu *et al*. developed mesoporous silica nanoparticle-resveratrol stacks and combined them with platelet-derived extracellular vesicles within complex hydrogels composed of gelatin methacrylate, silk fibroin glycidyl methacrylate, mesoporous silica nanoparticle-resveratrol, and platelet-derived extracellular vesicles. This study confirmed that the resveratrol formulation could effectively inhibit inflammation by suppressing the expression of iNOS and TNF-α [66]. This establish that it is possible to regulate inflammation by inhibiting the M1 polarization of macrophages and accelerating the transition of macrophages from M1 to M2 phenotype.

### 3.2 Natural Products that Promote the Polarization of M2 Macrophages

Medicinal plants also play a decisive role in the treatment of DFU by increasing the anti-inflammatory M2 polarization of macrophages. One of the primary bioactive components of *Ganoderma lucidum*, *Ganoderma lucidum* polysaccharide (GLP), has been utilized to treat a variety of illnesses due to its anti-inflammatory, anti-oxidant, fibroblast proliferation-promoting, antidiabetic, and other effects [67]. Research has demonstrated that GLP exerts an immunomodulatory effect, which prevents M1 macrophage polarization and encourages M2 macrophage polarization [68]. Li *et al.* prepared oxidized GLP/carboxymethyl chitosan/sodium alginate (OGLP-CMC/SA) hydrogel to observe its effect on diabetic wound repair [69]. Raw 264.7 cells were selected for *in vitro* evaluation, and CD86 and CD206 were utilized as M1 and M2 macrophage markers. It was discovered that the OGLP-CMC/SA hydrogel group expressed more CD206 and less CD86 than the control group. The findings imply that this might aid in the promotion of M2 polarization. Additionally, it was also found that the OGLP-CMC/SA hydrogel might boost IL-10 levels, lower TNF-α levels, and enhance cellular immune response. To assess the healing potential of OGLP-CMC/SA hydrogel, researchers created a diabetic wound model in mice. The findings demonstrated that *via* controlling macrophage polarization, granulation tissue formation, and collagen deposition, OGLP-CMC/SA hydrogel sped up the healing of diabetic wounds. Furthermore, it was discovered that the OGLP-CMC/SA hydrogel boosted the scavenging capacity and antibacterial activity of reactive oxygen species and fibroblast migration and proliferation.

*Poria cocos* has been widely used to treat diabetes and its complications [70]. A previous study revealed that *Poria cocos* (Fuling) targeted TGF-β/Smad7 associated collagen accumulation and enhanced nuclear factor erythroid 2-related factor 2-antioxidant mechanism to exert anti-skin aging effects in human dermal fibroblasts [71]. Hydrogels could protect and release therapeutic agents in a controlled and targeted manner in the diabetic wound environment. Hyaluronic acid further enhances cell adhesion, migration, and proliferation to promote cell function. Ding *et al.* proved that hyaluronic acid hydrogels (PTE-NES) prepared using a new oil-in-water nanoemulsion containing *Poria cocos* triterpene extract not only significantly accelerated diabetic wound healing, collagen deposition, M2 macrophage polarization, and angiogenesis, but also suppressed the inflammatory response in mouse experiments [72]. Their findings indicated that PTE-NEs reduced the differentiation of macrophages into the M1 pro-inflammatory type and induced the polarization of M2 pro-inflammatory macrophages. This reduction in local inflammation might potentially contribute to the acceleration of the wound-healing process.

Paeoniflorin (PF), an extract from *Paeonia lactiflora* roots, exhibits immunomodulatory properties by controlling the activity of macrophages [73, 74]. Yang *et al.* demonstrated that PF regulated the phenotype and function of macrophages by modulating the STAT signaling pathway. They developed a high molecular weight hyaluronic acid-based hydrogel to observe wound healing in diabetic mice after local administration [75]. In this study, a PF-loaded hyaluronic acid-based hydrogel (HA-PF) was prepared and stylized in a streptozotocin-induced diabetic mouse model. The results revealed that HA-PF significantly reduced the number of M1 phenotypes and increased the number of M2 phenotypes. Moreover, HA-PF down-regulated the release of pro-inflammatory factors, such as TNF-α, IL-1β, and iNOS, while up-regulating the expression of anti-inflammatory/pro-healing factors, including arginase 1, IL-10, and TGF-β. Furthermore, HA-PF enhanced the expression of CD31, VEGF, alpha-smooth muscle actin, and type I collagen. Evidence indicates that HA can modulate macrophage responses, which leads to the amelioration of inflammation, improved angiogenesis, enhanced re-epithelialization, and increased collagen deposition, thereby improving DFU.

Furthermore, resveratrol promoted M2 macrophage polarization, making it beneficial for both normal and diabetic wound repair [76]. Ding *et al.* investigated the modulation of macrophages as a pathway of diabetic wound healing [77]. These findings demonstrated that resveratrol induced the polarization of M2 macrophages and reduced the secretion of pro-inflammatory factors under the chronic inflammatory conditions associated with diabetes. Moreover, further investigations involving immunofluorescence revealed that resveratrol facilitated the healing of diabetic wounds by promoting M2 macrophage polarization *via* the activation of the PI3K/AKT signaling pathway.

Puerarin is a major bioactive ingredient isolated from the roots of *Pueraria lobata* (Willd) Ohwi and has been extensively used in the treatment of diabetes and diabetic complications [78]. Li *et al.* concluded that puerarin exhibited a substantial effect on wound healing in streptozotocin-induced diabetic models [79]. Additionally, puerarin has been found to potentially enhance the polarization of M2 macrophages, possibly *via* the inhibition of the NF-κB/ MAPK signaling axis.

Astragaloside IV (AS-IV), a compound found in *Astragali radix*, possesses potent anti-inflammatory and immunomodulatory properties [80]. Luo *et al.* investigated the effects of AS-IV on streptozotocin-induced wound healing in mice. The results revealed several positive outcomes associated with AS-IV treatment. First, mice receiving local injections of AS-IV exhibited a higher rate of wound closure than those treated with the vehicle alone [81]. In addition, AS-IV accelerated re-epithelialization, enhanced extracellular matrix deposition, and facilitated neovascularization, indicating its beneficial effects on diabetic wound healing. The researchers provide further supporting evidence suggesting that the upregulation of alternatively activated macrophages might be involved in the improvement of wound healing in AS-IV-treated diabetic mice.

Fu *et al.* investigated the impact of plant-derived quercetin on diabetic wound healing, given its known ability to expedite the healing process in general wounds [82]. The rats were treated with streptozotocin, and it was observed that quercetin effectively promoted wound contraction in a dose-dependent manner. Quercetin has been demonstrated to reduce inflammation, boost fibroblast activity, and stimulate collagen synthesis. The alleviation of inflammation has been attributed to its effects on macrophages. Quercetin significantly increased the number of CD208-positive cells and the levels of the M2 marker genes macrophage scavenger receptor 1, arginase 1, VEGF, and CD31 in the wound tissue of diabetic rats, as determined by immunohistochemical staining and quantitative real-time PCR. By modulating macrophage polarization from M1 to M2 phenotype, quercetin inhibited inflammation and facilitated the healing of diabetic wounds.

An extract derived from traditional Chinese medicine, emodin, has demonstrated its potential to reduce inflammation by increasing the proportion of M2 macrophages [83]. In this context, Chen *et al.* examined the mechanism underlying the ability of emodin to promote healing in db/db diabetic mice [84]. In comparison to vehicle-treated wounds, emodin was found to have a positive impact on wound healing, accelerating the healing time, facilitating neovascularization, and promoting ECM formation. Network pharmacology and transcriptome analyses suggested that the pro-healing effect of emodin on diabetic wounds may potentially be attributed to its downregulation or inhibition of the NF-κB signaling pathway. In both *in vitro* and *in vivo* experiments, emodin showed its ability to reduce inflammation and enhance the proportion of M2 macrophages. Western blotting revealed that emodin inhibited the phosphorylation of P65 in the NF-κB signaling pathway. Therefore, emodin holds promise as a beneficial agent for promoting wound healing by inhibiting the hyperactive NF-κB signaling pathway in diabetes and promoting polarization of M2 macrophages.

Qian *et al*. developed a new glycyrrhizic acid-based interpenetrating polymer network hybrid hydrogel, referred to as the light-enhanced interpenetrating polymer network hydrogel [85]. Research has indicated that the M2/M1 macrophage ratio was elevated in the silk fibroin/glycyrrhizic acid/inorganic Zn hydrogel group, accompanied by a reduction in M1 cytokine protein levels and an increase in M2 cytokine protein levels. These findings provide further evidence that silk fibroin/glycyrrhizic acid/inorganic Zn hydrogels effectively reduce the activation of M1 macrophages while promoting the transformation to the M2 phenotype.

Protocatechuic aldehyde extracted from *Salvia miltiorrhiza* has been recognized for its remarkable anti-inflammatory and antibacterial properties [86]. Fish gelatin is a compelling candidate for the formulation of hydrogel matrices owing to its excellent ability to induce anti-inflammatory transformation [87]. Recently, Fu *et al.* developed an all-natural hydrogel bandage, referred to as a chemical crosslinking network/fish gelatin/protocatechuic aldehyde [88]. This innovative hydrogel formulation has demonstrated the ability to alleviate inflammation by facilitating the conversion of pro-inflammatory M1 macrophages into anti-inflammatory M2 macrophages in mice, thereby promoting a more favorable wound-healing environment.

Similarly, other plant-derived components, such as ursolic acid (UA) and proanthocyanidins (PA), which are known for their low toxicity and multi-target functions, possess a variety of pharmacological activities, including hypoglycemic, antioxidant, and anti-inflammatory activities [89]. Lv and colleagues have recently designed a novel dressing composed of electrospun chitosan-polyvinyl alcohol nanofibers loaded with UA and demonstrated its experimental efficacy in promoting diabetic skin wound healing *via* macrophage modulation [90]. Furthermore, Wang *et al.* prepared a coacervate-based drug carrier consisting of oligochitosan and inositol hexaphosphate for the sustained release of PA [91]. The findings of these experiments demonstrated that UA and PA treatment leads to an increase in the proportion of M2:M1 macrophages accompanied by a reduction in the number of M1 macrophages. This implies that UA and PA potentially promote the transition from the pro-inflammatory phase to the pro-healing phase in diabetic wound healing by regulating the phenotypic transition of macrophages from M1 to M2.

In addition, Chlorogenic Acid (CA) from coffee and blueberries is also used to develop a triple crosslinked hydrogel called oxidized hyaluronic acid-PBAs-PLL@Zinc-CA@ epidermal growth factor [92]. When applied to the wounds of mice, this hydrogel could markedly speed up wound contraction, decrease inflammation, promote the creation of granulation tissue, thicken the epithelium, and deposit collagen. Subsequent experimental research revealed that the hydrogel's promotion of macrophage transformation from M1 to M2 through the NF-κB/JAK-STAT signaling pathway successfully regulated the *in vivo* inflammatory milieu, accounting for the wound healing mechanism. Given the substantial potential of curcumin to accelerate the healing process of diabetic wounds, a novel poly (vinyl alcohol)-chitosan/sodium alginate-cur hydrogel has been engineered, incorporating curcumin to enhance its therapeutic efficacy [93]. Further research demonstrated that this hydrogel might induce the M1 to M2 transition, eliminate reactive oxygen species, suppress IL-1β, enhance CD31 expression, induce angiogenesis and collagen deposition, and significantly speed up the healing process from wounds.

### 3.3 Natural Products that Strengthen Macrophage Function

Numerous studies have reported the potential benefits of papain, an enzyme derived from the ripe fruit of *Carica papaya* Linn, in wound debridement. Additionally, *C. papaya* seed extracts also appeared to promote wound healing in rats [94, 95]. Based on this, Collard *et al.* investigated the effects of fermented papaya on DFU and its underlying mechanism [96]. The study revealed that fermented papaya significantly improved wound closure in diabetic mice. Further investigation indicated that fermented papaya enhanced the recruitment of macrophages to the wound site, directly improved nitric oxide production, elevated iNOS gene expression in the wound tissue, increased the level of superoxide, indirectly promoted angiogenesis, and eventually promoted diabetic wound healing.

Hsian-tsao (*Mesona procumbens* Hemsl.) has been identified as a potential therapeutic agent for accelerating wound healing. To assess the effects of hsian-tsao extracts and their polysaccharides on impaired DFU, Fan *et al.* used methylglyoxal-induced cells and streptozotocin-induced mice as diabetes-like experimental models [97]. This study indicated that the ethanol extract and water extract of hsian-tsao exhibited positive effects on diabetic wound healing. These effects were observed through a reduction in crust formation and enhanced granulation, angiogenesis, and re-epithelialization. The inhibitory effect of hsian-tsao extracts and their polysaccharides on macrophage dysfunction has been identified as one of the mechanisms contributing to accelerated wound healing. Both the ethanol extract and water extract of hsian-tsao reduced reactive oxygen species production in methylglyoxal-induced RAW 264.7 cells and inhibited the expression of inflammation-related factors in methylglyoxal-induced RAW 264.7 macrophages. Furthermore, the ethanol extract and water extract of hsian-tsao strengthened the phagocytosis of *Staphylococcus aureus* and *Pseudomonas aeruginosa* in MG-treated macrophages, potentially aiding the improvement of impaired wound healing by inhibiting bacterial infections.

## CONCLUSION AND FUTURE PROSPECTS

Multiple pieces of evidence strongly suggest that medicinal plants and bioactive compounds derived from plants display substantial potential to accelerate DFU healing. Recognizing the pivotal role of macrophages in chronic diabetic wounds, numerous studies have highlighted the promising effects of medicinal plants in fostering the M2 polarization of macrophages, suppressing M1 polarization, and enhancing their reparative functions. A particularly encouraging aspect is the minimal toxicity demonstrated by these medicinal plants, as supported by available evidence.

Nonetheless, further research is imperative to meticulously evaluate extracts that exhibit the best efficacy for wound healing to mitigate the risks of adverse effects and unintended consequences. Furthermore, it is worth noting that the current body of research is predominantly confined to animal experiments, with limited in-depth investigations into the precise regulatory effects of medicinal plants on macrophages and even fewer clinical trials exploring these effects. The introduction of compounds, such as ON101, serves as a prime illustration of this research direction, and it is anticipated that further research will lead to the development of drugs with enhanced efficacy for DFU treatment. Hence, well-structured and sufficiently powered randomized controlled trials involving human populations are imperative to establish the scientific validity of the beneficial effects of medicinal plants on DFU. Simultaneously, there is a pressing need for greater emphasis on uncovering the mechanisms through which medicinal plants exert their effects on DFU, as this aspect has garnered relatively less attention within the research community. Notably, the advent of innovative technologies and methodologies, such as genomics, has provided valuable support for identifying potential targets and elucidating the mechanisms of action underlying the effects of medicinal plants on DFU.

The healing process in DFU is typically orchestrated by the release of growth factors and cytokines, which are generated by various cells activated as part of the immune response. Within this response, there is a transition in the macrophage phenotype from pro-inflammatory M1 to anti-inflammatory M2 macrophages, ultimately steering the course of wound healing. However, in clinically challenging cases of DFU, this shift in macrophage function is not easily achieved, and macrophages tend to linger in a pro-inflammatory state, leading to chronic inflammation and an inadequate shift toward M2 macrophages. Therefore, there is a need for further exploration of the mechanisms by which medicinal plants and plant-derived bioactive compounds or derivatives facilitate the rebalancing of macrophage polarization in patients with refractory DFU. Given the complexity of macrophages in regulating the healing of diabetic foot ulcers, it is essential to consider the interactions between macrophages and other cells. Studies have demonstrated that the cooperation of macrophages and mast cells (MCs) might contribute to cartilage damage and repair, the mechanism of which triggers the production of MMP and activates the M1 macrophage secretion factor. MCs can encourage the breakdown of the cartilage matrix [98]. In the inflammatory stage, MCP-1 secreted by MCs promotes monocytes recruited from blood to convert into macrophages, thereby secreting cytokines and engaging in wound healing jointly. MCs also recruit monocytes through secretion media, thus secreting cytokines, which are used as signals to activate fibroblasts [99]. The dysregulation of macrophages and neutrophils plays a key role in DFU healing, and their dysregulation and dysfunction further lead to infection control defects and healing damage [100]. Macrophages also interact with platelets [101], stem cells [22], and others during the healing process. Comprehending the dynamic between macrophages and other cells holds immense importance in investigating the mechanisms of wound healing and developing novel therapeutic interventions.

Currently, systemic delivery methods, in which drugs administered to patients with diabetes enter the bloodstream, are widely employed. Providing treatment for DFU is crucial because the absence of treatment can lead to severe or even causative side effects. Therefore, medicinal plants play a vital role in the topical treatment of DFU. However, owing to their inherent characteristics, such as drug stability and delivery, medicinal plants often entail a time-consuming and delayed response in the context of DFU. On the other hand, drug delivery *via* nanoparticles and nanomaterials in diabetic wound healing treatments offers several advantages, particularly in expediting the wound healing process. Nanoparticles serve as effective drug delivery systems with precise control of drug release. Notably, when combined with hydrogels, nanoparticles enable controlled drug release, localization at the wound site, and structural remodeling. Hence, nanoparticles function as carriers for the delivery of drugs derived from medicinal plants or natural products with potent anti-inflammatory properties.

## Figures and Tables

**Fig. (1):**
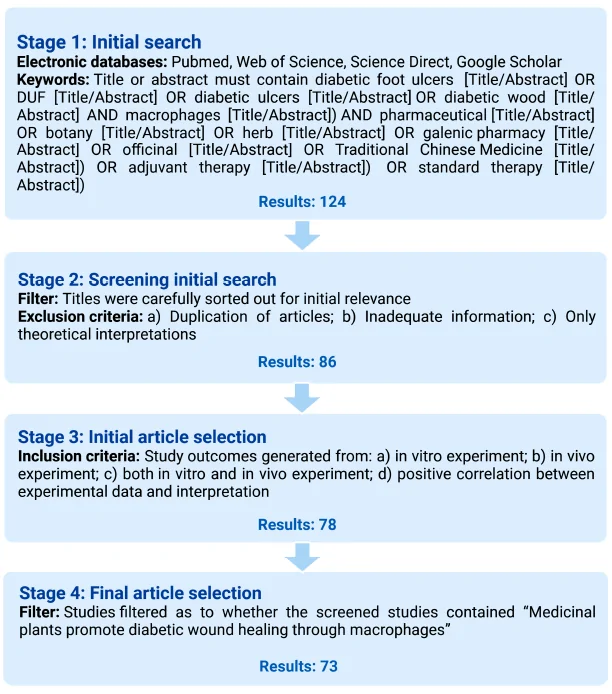
The flowchart depicting literature screening.

**Fig. (2):**
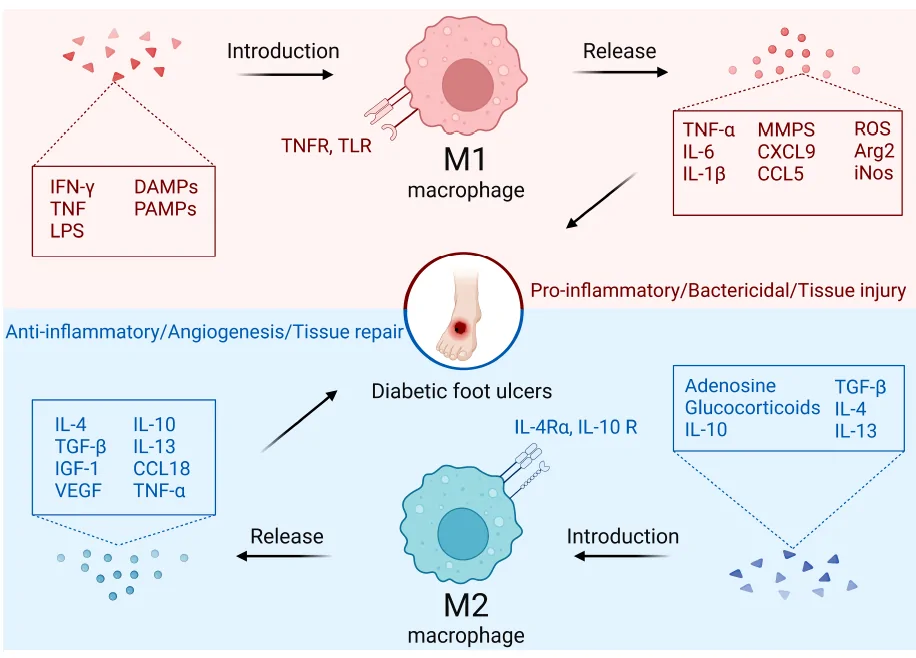
Polarization of macrophages in DFU. Macrophages undergo polarization, resulting in the emergence of two types: M1 macrophages and M2 macrophages. M1 macrophages are induced by IFN-γ, TNF, and LPS and release ROS, proinflammatory cytokines (*e.g.*, IL-1β, IL-6, and TNF-α), free radicals (iNOS), and chemokines (*e.g.*, CXCL9). M2 macrophages are induced by TGF-β, IL-4, and IL-13 and release anti-inflammatory cytokines (*e.g.*, IL-4), chemokines (*e.g.*, CCL18), and growth factors (VEGF, TGF-β). The M1 macrophages are pro-inflammatory and bactericidal, while the M2 macrophages act to switch off inflammation and regulate re-vascularization and wound repair. Arg-2, arginase 2; CCL, chemokine (C-C motif) ligand; CXCL, chemokine (C-X-C motif) ligand; DAMPs, damage-associated molecular patterns; IFN-γ, interferon-gamma; IGF-1, insulin-like growth factor 1; IL, interleukin; IL4Rα, interleukin-4 receptor subunit alpha; iNOS, inducible nitric oxide synthase; LPS, lipopolysaccharide; MMPS, matrix metalloproteinases; PAMPs, pathogen-associated molecular patterns; ROS, reactive oxygen species; TGF, transforming growth factor; TLR, Toll-like receptor; TNF, tumor necrosis factor; TNFR, tumor necrosis factor receptor; VEGF, vascular endothelial growth factor.

**Fig. (3):**
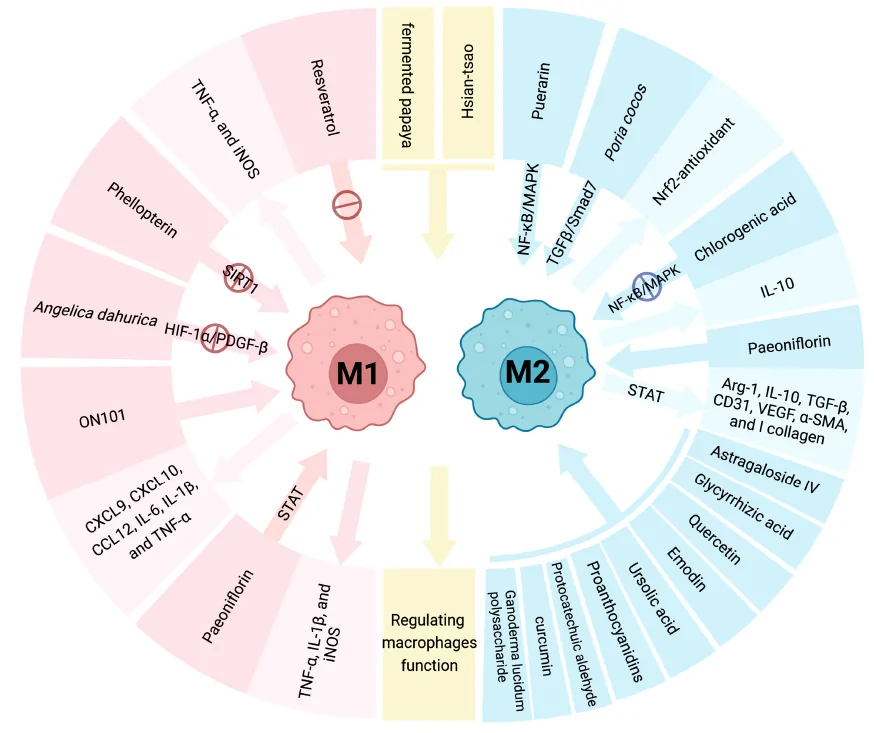
Signal pathways of medicinal plants promoting DFU healing *via* macrophage polarization. ON101 effectively attenuates the polarization of M1 macrophages, which downregulates M1-related chemokines (CXCL9, CXCL10, and CCL12) and cytokines (IL-6, IL-1β, and TNF-α). *Angelica dahurica* accelerates wound healing *via* the HIF-1α/PDGF-β signaling pathway. Phellopterin, a monomeric compound derived from *Angelica dahurica*, exerts an anti-inflammatory effect that facilitates diabetes-associated cutaneous wound healing *via* the involvement of SIRT1. The formulation of resveratrol inhibits the M1 polarization of macrophages and inflammation by suppressing the expression of iNOS and TNF-α. Paeoniflorin regulates the phenotype and function of macrophages by modulating the STAT signaling pathway and down-regulating the release of pro-inflammatory factors, such as TNF-α, IL-1β, and iNOS. Puerarin enhances the polarization of M2 macrophages, possibly *via* the inhibition of the NF-κB/MAPK signaling axis. *Poria cocos* targets TGFβ/Smad7-associated collagen accumulation and enhances the Nrf2-antioxidant mechanism to exert anti-skin aging effects in human dermal fibroblasts. Chlorogenic acid promotes M2 polarization by inhibiting NF-κB/JAK-STAT signaling pathway and upregulates IL-10. Paeoniflorin regulates both the phenotype and function of macrophages by modulating the STAT signaling pathway, up-regulating the expression of anti-inflammatory/pro-healing factors, including arginase 1, IL-10, and TGF-β, and enhancing the expression of CD31, VEGF, α-SMA, and type I collagen. Astragaloside IV, glycyrrhizic acid, quercetin, emodin, ursolic acid, proanthocyanidins, protocatechuic aldehyde, curcumin, and *Ganoderma lucidum* polysaccharide promote the transition from the pro-inflammatory phase to the pro-healing phase in diabetic wound healing by regulating the phenotypic transition of macrophages from M1 to M2. Fermented papaya and Hsian-tsao impair wound healing by regulating macrophage function. **Abbreviations: ** α-SMA, alpha-smooth muscle actin; CCL, chemokine (C-C motif) ligand; CD, cluster of differentiation; CXCL, chemokine (C-X-C motif) ligand; IL, interleukin; iNOS, inducible nitric oxide synthase; Nrf2, nuclear factor erythroid 2-related factor 2; SIRT1, silent information regulator 2 homolog 1; STAT, signal transducer and activator of transcription; TGF, transforming growth factor; VEGF, vascular endothelial growth factor.

**Table 1:** Differing biological and physiological features of M1 and M2 macrophages phenotypes.

**Phenotype**	**M1**	**M2**			
**Classification**		**M2a**	**M2b**	**M2c**	**M2d**
Stimuli	LPS/IFN-γ/TNFα/GM-CSF	IL-4/IL-13	Immune complexes/TLR agonists/IL-1β agonists	Glucocorticoids/IL-10/ TGF-β	TLR agonists/AR agonists
Cell expression markers	CD80/CD86/HLA-DR /TLR-4/iNOS	CD163/CD206/CD369	CD86/HLA-DR/TNF sf14	CD150/CD163/CD206 /CCR2/TLR-1	iNOS
Cytokines, chemokines, and other secreted mediators	TNF-α/IL-1β/IL-6/IL-12/ IL-23/IL-27/CXCL9/CXCL10/ CXCL11/CXCL16/CCL5	IL-10/TGF-β/CCL17/ CCL18/CCL22/CCL24	TNF-α/IL-1β/ IL-6/IL-10/CCL1	IL-10/TGF-β/CCL16/CCL18/ CXCL13	IL-10/VEGF
Functions	Pro-inflammatory, Th1 response, Tumor resistance	Anti-inflammatory, tissue remodeling	Th2 activation, immunoregulation	Phagocytosis of apoptotic cells	Angiogenesis, tumor progression

**Abbreviations:** AR, adenosine receptor; CCL, chemokine (C-C motif) ligand; CD, cluster of differentiation; CXCL, chemokine (C-X-C motif) ligand; GM-CSF, granulocyte macrophage-colony stimulating factor; HLA-DR, human leukocyte antigen-DR; IFN-γ, interferon-gamma; IL, interleukin; iNOS, inducible nitric oxide synthase; LPS, lipopolysaccharides; TGF, transforming growth factor; Th1, T helper 1 cell; Th2, T helper 2 cell; TLR agonists, toll-like receptors agonists; TNF-α, tumor necrosis factor alpha; TNF sf14, tumor necrosis factor superfamily member 14.

**Table 2:** The mechanisms of medicinal plants and product promoting DFU healing *via* macrophages.

**Plant Product/ Plant**	**Name of the Compound**	**Animal Modeling**	**Effects on Diabetic Wounds**	**Mechanism on Diabetic Wounds**	**Research Type**	**References**
ON101/ *Plectranthus amboinicus* and *Centella asiatica*	Carvacrol, thymol, β-caryophyllene, α-humulene.	High-fat diet -induced (for 10 weeks) diabetes and db/db mice	Rate of wound recovery↑ Granulation tissue growth and epithelialization↑	iNOS expressions↓ The number of CD163 cells and CD206 cells↑	Animal research, cell experiment	[57]
FPP/ *Carica papaya* Linn	Carpaine, violaxanthin, rennin, cryptoxanthin	db/db mice	Wound closure↑ Angiogenesis↑	NO, CD31 and VEGF level↑ Superoxide levels in macrophages↑	Animal research	[96]
*Angelica dahurica*	Coumarins, volatile oils	db/db mice	Rate of wound closure↑ Inflammatory cell infiltration↓ Neovascularization, granulation tissue formation↑	CD68, IL-1β, TNF-α, iNOS, and IL-6 expression↓ Arg-1, VEGF and TGF-β1 expression↑	Animal research	[60]

**Abbreviations:** Arg-1, arginase 1; CD, cluster of differentiation; FPP, fermented papaya preparation; IL, interleukin; iNOS, inducible nitric oxide synthase; NO, nitrous oxide; ON101, a topical cream formulated using identified active extracts from *Plectranthus amboinicus* and *Centella asiatica*; STZ, streptozocin; TGF, transforming growth factor; VEGF, vascular endothelial growth factor.

**Table 3:** The mechanisms of phytochemical constituents in medicinal plants promoting DFU healing *via* macrophages.

**Name of the Compound**	**Carrier**	**Animal Modeling**	**Effects on Diabetic Wounds**	**Mechanism on Diabetic Wounds**	**Research Type**	**References**
Resveratrol	GelMA/SFMA/MSN-RES/PDEVs	db/db mice	Wound closure↑ Angiogenesis, and collagen deposition↑	TGF-β1 and Arg-1 expression↑ TNF-α and iNOS expression↓	Animal research, cell experiment	[66]
*Ganoderma lucidum* polysaccharide	OGLP-CMC/SA	STZ-induced diabetic rats (50 mg/kg for 5 days)	Rate of wound healing↑ the growth of granulation tissue↑ Inflammatory infiltration↓ Collagen deposition and angiogenesis↑	CD206↑	Animal research, cell experiment	[69]
*Poria cocos* triterpenes	PTE-NEs	STZ-induced diabetic rats (65 mg/kg)	Rate of wound closure↑ Inflammatory infiltration↓ Collagen deposition and angiogenesis↑	IL-1β, TNF-α and IL-6 expression↓ CD31, α-SMA, VEGF and Collagen I expression↑	Animal research, cell experiment	[72]
Paeoniflorin	HA-PF	STZ-induced diabetic rats (55 mg/kg for 5 days)	The wound closure↑ Inflammatory infiltration and epidermal hyperplasia↓ Angiogenesis and collagen deposition↑	Pro-inflammatory factors (iNOS, TNF-α, and IL-1β)↓ Anti-inflammatory/prohealing factors (Arg-1, IL-10, and TGF-β)↑ CD31, VEGF, α-SMA and type I collagen expression↑	Animal research, cell experiment	[75]
Resveratrol	-	STZ-induced diabetic rats (50 mg/kg)	The wound closure↑ Tissue inflammatory↓ Re-epithelialization and collagen deposition↑	α-SMA, Collagen I, Arg-1 and CD206 expression↑ The secretion of inflammatory factors (TNF-α, iNOS and IL-1β)↓	Animal research, cell experiment	[77]
Puerarin	-	STZ-induced diabetic rats (50 mg/kg for 5 days)	Wound areas↓ Inflammatory cell infiltration↓ The state of collagen disorders, epithelial abnormalities and structural alterations↑	TNF-α, IL-1β and Cd11b expression level↓ Arg-1, IL-10, TGF-β1, CD206 and CD163 expression↑	Animal research, cell experiment	[79]
*Astragali radix*	-	STZ-induced diabetic rats (40 mg/kg for 5 days)	Rate of wound closure↑ Neovascularization and re-epithelialization↑ ECM deposition↑	The expression of arginase-1 and Ym1 and F4/80 CD206 cells ↑	Animal research	[81]
Quercetin	-	STZ-induced diabetic rats (60 mg/kg)	Rate of wound contraction↑ Inflammatory cells↓ Fibroblast activity and collagen deposition↑	CD31, VEGF-α, IL-10, Msr-1 and Arg-1 expression level↑ IL-6, TNF-α, iNOS expression level↓	Animal research	[82]
Emodin	-	db/db mice	Wound healing time↓ Neovascularization and ECM formation↑	CD31, PCNA, α-SMA and VEGF expression↑ Arg-1, Retnla, and Clec10a expression↑ TNF-α and IL-6 expression↓	Animal research, cell experiment	[84]
Glycyrrhizic acid	SF/GA/Zn	STZ-induced diabetic rats	Rate of wound closure↑ Inflammatory cell infiltration↓ Neovascularization and collagen deposition↑ CD31 and VEGF expressions↑	TNF-α and Myeloperoxidase expression level↓	Animal research, cell experiment	[85]
Protocatechuic aldehyde	FGMA/FG/PA	STZ-induced diabetic rats	Rate of wound closure↑ Neovascularization, collagen deposition and hair follicle regeneration↑ Myeloperoxidase and matrix metalloproteinase 9 expressions↓	CD31, α-SMA and VEGF expression↑	Animal research, cell experiment	[88]
Ursolic acid	CS-PVA-UA	STZ-induced diabetic rats (65 mg/kg for 5 days)	Rate of wound contraction↑ The Inflammation↓ The re-epithelialization collagen deposition, angiogenesis and Regeneration of Hair Follicles↓	-	Animal research, cell experiment	[90]
Proanthocyanins	ADC-PA	db/db mice	Rate of wound closure↑ Collagen deposition and re-epithelialization↑	-	Animal research, cell experiment	[91]
EE and WEP	-	STZ-induced diabetic rats (65 mg/kg for 7 days)	Wound area↓ Inflammation↓ Granulation and re-epithelialization↑	-	Animal research, cell experiment	[97]
Chlorogenic acid	OHA-PP@Z- CA@EGF	STZ-induced diabetic (60 mg/kg)	Wound inflammation ↓ Granulation tissue formation, epithelial thickness, and collagen deposition↑	NO, TNF-α, IL-1β, IL-6, iNOs, ROS, and COX-2 expression↓ IL-10 levels↑	Animal research, cell experiment	[92]
Curcumin	PCSA	STZ-induced diabetic (60 mg/kg)	Rate of wound closure↑ angiogenesis and collagen deposition↑	IL-1β expressions↓ CD31 expressions↑	Animal research, cell experiment	[93]

**Abbreviations:** ADC-PA, a complex coacervate-based drug carrier- proanthocyanins; α-SMA, alpha-smooth muscle actin; Arg-1, arginase 1; Cd11b, CD11 antigen-like family member B; CD, cluster of differentiation; Clec10a, C-type lectin domain family 10, member a; CS-PVA-UA, chitosan/polyvinyl alcohol/ursolic acid; EE, ethanol extracts of hsian-tsao; ECM, extracellular matrix; F4/80, EGF-like module-containing mucin-like hormone receptor-like 1;FGMA/FG/PA, a chemical crosslinking network/fish gelatin/protocatechuic aldehyde; GelMA/SFMA/MSN-RES/PDEVs, gelatin methacrylate/silk fibroin glycidyl methacrylate/mesoporous silica nanoparticles-resveratrol/platelet-derived extracellular vesicles; HA-PF, hyaluronic acid/paeoniflorin; IL, interleukin; iNOS, inducible nitric oxide synthase; MCP-1, monocyte chemoattractant protein-1; Msr-1, Macrophage scavenger receptor 1; NO, nitrous oxide; OGLP-CMC/SA, oxidized *Ganoderma lucidum* polysaccharide/carboxymethyl chitosan/sodium alginate; OHA-PP@Z-CA@EGF, oxidized hyaluronic acid-ε-polylysine@ZIF-8@epidermal growth factor; PCNA, proliferating cell nuclear antigen; PCSA, poly(vinyl alcohol) (PVA)-chitosan (CS)/sodium alginate (SA)-Cur; PTE-NEs, *poria cocos* triterpenes extract/nanoemulsions; Retnla, resistin-like alpha; SF/GA/Zn, silk fibroin/glycyrrhizic acid/zn; STZ, streptozocin; TGF-β1, transforming growth factor beta1; TNF-α, tumor necrosis factor alpha; VEGF, vascular endothelial growth factor; WEP, water extracts of hsian-tsao; Ym1, chitinase 3-like 3.

## LIST OF ABBREVIATIONS

AS-IV: Astragaloside IV
CCL: Chemokine (C-C Motif) Ligand
CD: Cluster of Differentiation
CXCL: Chemokine (C-X-C Motif) Ligand
DFU: Diabetic Foot Ulcer
ECM: Extracellular Matrix
HA: Hyaluronic Acid
IL: Interleukin
iNOS: Inducible Nitric Oxide Synthase
LPS: Lipopolysaccharide
MCs: Mast Cells
OGLP-CMC/SA: Oxidized *Ganoderma lucidum* Polysaccharide /Carboxymethyl Chitosan/Sodium Alginate
PA: Proanthocyanidins
PF: Paeoniflorin
STAT: Signal Transducer and Activator of Transcription
TNF-α: Tumor Necrosis Factor Alpha
TNFR: Tumor Necrosis Factor Receptor
UA: Ursolic Acid
VEGF: Vascular Endothelial Growth Factor

## DECLARATION

The figures were created from the resources by BioRender.com (https://app.biorender.com).
